# Management of Asymptomatic Sporadic Nonfunctioning Pancreatic Neuroendocrine Neoplasms (ASPEN) ≤2 cm: Study Protocol for a Prospective Observational Study

**DOI:** 10.3389/fmed.2020.598438

**Published:** 2020-12-23

**Authors:** Stefano Partelli, John K. Ramage, Sara Massironi, Alessandro Zerbi, Hong Beom Kim, Patricia Niccoli, Francesco Panzuto, Luca Landoni, Ales Tomazic, Toni Ibrahim, Gregory Kaltsas, Emilio Bertani, Alain Sauvanet, Eva Segelov, Martyn Caplin, Jorgelina Coppa, Thomas Armstrong, Martin O. Weickert, Giovanni Butturini, Stefan Staettner, Florian Boesch, Mauro Cives, Carol Anne Moulton, Jin He, Andreas Selberherr, Orit Twito, Antonio Castaldi, Claudio Giovanni De Angelis, Sebastien Gaujoux, Hussein Almeamar, Andrea Frilling, Emanuel Vigia, Colin Wilson, Francesca Muffatti, Raj Srirajaskanthan, Pietro Invernizzi, Andrea Lania, Wooil Kwon, Jacques Ewald, Maria Rinzivillo, Chiara Nessi, Lojze M. Smid, Andrea Gardini, Marina Tsoli, Edgardo E. Picardi, Olivia Hentic, Daniel Croagh, Christos Toumpanakis, Davide Citterio, Emma Ramsey, Barbara Mosterman, Paolo Regi, Silvia Gasteiger, Roberta E. Rossi, Valeria Smiroldo, Jin-Young Jang, Massimo Falconi

**Affiliations:** ^1^Pancreatic Surgery Unit, Pancreas Translational & Clinical Research Center, IRCCS San Raffaele Scientific Institute, Milan, Italy; ^2^Kings Health Partners NET Center, Kings College Hospital London, London, United Kingdom; ^3^Division of Gastroenterology, Centre for Autoimmune Liver Diseases, Department of Medicine and Surgery, University of Milano-Bicocca, Monza, Italy; ^4^Humanitas Clinical and Research Center – IRCCS, Rozzano, Italy; ^5^Department of Surgery, Seoul National University Hospital, Seoul National University College of Medicine, Seoul, South Korea; ^6^Department of Medical Oncology, Paoli-Calmettes Institute, Marseille, France; ^7^Digestive Disease Unit, ENETS Center of Excellence, Sant' Andrea University Hospital, Rome, Italy; ^8^Department of Surgery, Pancreas Institute, Verona ENETS Center of Excellence, University and Hospital Trust of Verona, Verona, Italy; ^9^Department of Abdominal Surgery and Gastroenterology and Hepatology, Faculty of Medicine, University Medical Centre Ljubljana, University of Ljubljana, Ljubljana, Slovenia; ^10^Osteoncology and Rare Tumors Center, Istituto Scientifico Romagnolo per lo Studio e la Cura dei Tumori (IRST) IRCCS, Meldola, Italy; ^11^First Department of Propaedeutic and Internal Medicine, Laiko University Hospital, National and Kapodistrian University of Athens, Athens, Greece; ^12^Division of Gastrointestinal Surgery, European Institute of Oncology, Milan, Italy; ^13^Department of HPB Surgery and Liver Transplantation and Pancreatology, Beaujon Hospital, University Paris 7 Denis Diderot, Assistance publique-Hôpitaux de Paris, 100, Boulevard du Général-Leclerc, Clichy, France; ^14^Department of Oncology and Surgery (School of Clinical Sciences at Monash Health), Monash University, Clayton, VIC, Australia; ^15^Centre for Gastroenterology, Neuroendocrine Tumour Unit, ENETS Centre of Excellence, Royal Free Hospital, London, United Kingdom; ^16^Gastrointestinal and Hepato-Pancreatic Surgery and Liver Transplantation Unit, Fondazione, IRCCS Istituto Nazionale Tumori (INT, National Cancer Institute) and Università degli Studi di Milano, Milan, Italy; ^17^Department of Hepatobiliary Surgery, Wessex NET Group ENETS Centre of Excellence, University Hospital Southampton, Southampton, United Kingdom; ^18^The ARDEN NET Centre, European Neuroendocrine Tumour Society (ENETS) Centre of Excellence (CoE), University Hospitals Coventry and Warwickshire NHS Trust, Coventry, United Kingdom; ^19^Department of Surgery, Pederzoli Hospital, Peschiera del Garda, Italy; ^20^Department of General, Visceral and Vascular Surgery, Salzkammergutklinikum Vöcklabruck, Vöcklabruck, Austria; ^21^Department of General, Visceral and Transplantation Surgery, University Hospital, LMU Munich, Munich, Germany; ^22^Section of Medical Oncology, Department of Biomedical Sciences and Clinical Oncology (DIMO), University of Bari 'Aldo Moro', Bari, Italy; ^23^Division of General Surgery, University of Toronto, Toronto, ON, Canada; ^24^Department of Surgery, University Health Network, Princess Margaret Cancer Centre, University of Toronto, Toronto, ON, Canada; ^25^Department of Surgery, The Sol Goldman Pancreatic Cancer Research Center, Johns Hopkins Medical Institutions, Baltimore, MA, United States; ^26^Section Endocrine Surgery, Division of General Surgery, Department of Surgery, Medical University, Vienna, Austria; ^27^Sackler Faculty of Medicine, Endocrine Institute, Meir Medical Center, Tel-Aviv University, Tel-Aviv, Israel; ^28^Department of Clinical Medicine and Surgery, University of Naples Federico II, Naples, Italy; ^29^Gastroenterology Unit, Department of Medical Sciences, City of Health and Science Hospital, Turin, Italy; ^30^Department of Digestive, Hepatobiliary and Endocrine Surgery, Paris Descartes University, Cochin Hospital, Paris, France; ^31^National NET Centre and ENETS Centre of Excellence, St Vincent's University Hospital, Dublin, Ireland; ^32^Department of Surgery and Cancer, Imperial College London, London, United Kingdom; ^33^Centro Hepatobiliopancreático, Hospital Curry Cabral, Nova Univerditu of Lisbon, Lisbon, Portugal; ^34^HPB Surgical Unit, Newcastle upon Tyne Teaching Hospitals Foundation Trust, Newcastle upon Tyne, United Kingdom; ^35^General and Oncologic Surgery Unit, Morgagni-Pierantoni Hospital, Forlì, Italy; ^36^Department of Visceral, Transplantation and Thoracic Surgery, Medical University of Innsbruck, Innsbruck, Austria; ^37^Department of Pathophysiology and Transplantation, University of Milan, Milano, Italy

**Keywords:** small nonfunctioning pancreatic neuroendocrine neoplasm, NF-PanNEN_2 cm, management, surgery, surveillance, follow-up, ASPEN study

## Abstract

**Introduction:** The optimal treatment for small, asymptomatic, nonfunctioning pancreatic neuroendocrine neoplasms (NF-PanNEN) is still controversial. European Neuroendocrine Tumor Society (ENETS) guidelines recommend a watchful strategy for asymptomatic NF-PanNEN <2 cm of diameter. Several retrospective series demonstrated that a non-operative management is safe and feasible, but no prospective studies are available. Aim of the ASPEN study is to evaluate the optimal management of asymptomatic NF-PanNEN ≤2 cm comparing active surveillance and surgery.

**Methods:** ASPEN is a prospective international observational multicentric cohort study supported by ENETS. The study is registered in ClinicalTrials.gov with the identification code NCT03084770. Based on the incidence of NF-PanNEN the number of expected patients to be enrolled in the ASPEN study is 1,000 during the study period (2017–2022). Primary endpoint is disease/progression-free survival, defined as the time from study enrolment to the first evidence of progression (active surveillance group) or recurrence of disease (surgery group) or death from disease. Inclusion criteria are: age >18 years, the presence of asymptomatic sporadic NF-PanNEN ≤2 cm proven by a positive fine-needle aspiration (FNA) or by the presence of a measurable nodule on high-quality imaging techniques that is positive at ^68^Gallium DOTATOC-PET scan.

**Conclusion:** The ASPEN study is designed to investigate if an active surveillance of asymptomatic NF-PanNEN ≤2 cm is safe as compared to surgical approach.

## Introduction

Nonfunctioning pancreatic neuroendocrine neoplasms (NF-PanNEN) are rare tumors that exhibit a wide heterogeneity of aggressiveness. The current World Health Organization (WHO) classification identified three categories of NF-PanNEN (NF-PanNEN-G1, NF-PanNEN-G2, and NF-PanNEN-G3) based on Ki-67 value (1). Indications for surgery include the presence of a localized NF-PanNEN in the absence of distant metastases as curative resection of these tumors is associated with favorable prognosis especially for low grade disease (2–4). In the last decade a dramatic increase in diagnosis of small, incidentally discovered, NF-PanNEN has been observed (5–7). Several studies have highlighted the role of incidental diagnosis as a powerful prognostic factor for NF-PanNEN (8, 9). Moreover, other investigators have observed a clear relationship between the tumor diameter and the risk of malignancy and systemic progression (10–12). In particular, a tumor size ≤2 cm seems to be associated with a negligible risk of disease recurrence after surgery and to a very low incidence of aggressive features such as lymph node involvement (4, 13). On this basis, the European Neuroendocrine Tumor Society (ENETS) guidelines suggest that a “*wait and see*” approach for small asymptomatic NF-PanNEN may be advocated (2, 14) The safety of a conservative management for these entities have been explored in several experiences (15–21). All these studies have confirmed that an intensive surveillance for small incidental NF-PanNEN is safe since none of the patients in the observational group deceased for disease and the appearance of distant metastases during follow-up has been reported only for those patients with lesions lager than 2 cm (20). Nevertheless, available data are based only on retrospective series with a significant heterogeneity of inclusion criteria and different tumor diameter *cut-off* s (15–19). Moreover, some authors still consider surgery the most effective treatment also for these apparently indolent tumors (22). Aim of the present study is to evaluate the most appropriate management of sporadic asymptomatic NF-PanNEN ≤2 cm.

## Methods

### Study Aim

The ASPEN study aims to determine the best management for small, nonfunctioning, asymptomatic NF-PanNEN ≤2 cm comparing active surveillance (AS) and surgical resection (SR). The hypothesis is that AS is a safe approach that prevents unnecessary surgery in a considerable number of cases thus avoiding surgical-related morbidity and mortality.

### Study Design and Setting

The study is designed as a prospective international observational multicentric cohort study, coordinated by the Pancreatic Surgery Unit and Pancreas Translational & Clinical Research Center at San Raffaele Scientific Institute, Milan, Italy (Lead Study Centre) under the auspices of the European Neuroendocrine Tumor Society (ENETS). In total, 41 centers from 16 countries (Australia, Austria, Canada, Italy, France, Germany, Greece, Ireland, Israel, Netherlands, Portugal, Slovenia, Spain, South Korea, United Kingdom, United States) are actively participating in the trial. The study duration is 6 years, ethical committee of the Lead Study Center approved the study in June 2017 and patients are being recruited for 5 years from August 2017 to August 2022, with a follow-up of 1 year at least (end of the study: July 2023). The ASPEN study is registered in ClinicalTrials.gov with the identification code: NCT03084770. Participating study centers identify, recruit patients and send pseudonymized data to the lead center, which is responsible for statistical analysis, storing and controlling data. The research database will be managed and analyzed by the Lead Study center research team.

### Primary Endpoint

The primary endpoint is disease/progression-free survival, defined as the time from study enrolment to the first evidence of progression (AS group) or recurrence of disease (SR group) or death from disease.

### Secondary Endpoints

Secondary endpoints are: (i) to evaluate the frequency of asymptomatic sporadic NF-PanNEN ≤2 cm among overall sporadic NF-PanNEN. For this purpose, participating centers are required to give yearly the number of patients with NF-PanNEN referred to their institution, (ii) to analyze the outcome of patients with an indication for surgical resection, in terms of number of operated patients, surgical procedures, morbidity, mortality, and NF-PanNEN recurrence after surgery, (iii) to evaluate NF-PanNEN evolution, in terms of development of symptoms, tumor growth, development of distant metastases and secondary pancreatic duct dilatation, (iv) to measure the perceived burden of surveillance or follow-up after surgery for participants, as assessed by questionnaires regarding attitude toward surveillance and general anxiety and depression [Hospital Anxiety and Depression scale, HADS (23), EORTC QLQ-C30-version 3 (24) and EORTC QLQ-GI.NET21 Module (25)].

### Sample Size

The reported incidence rate of PanNEN is 0.4/100.000 inhabitants (5, 7) considering that rate of NF-PanNEN with a diameter ≤2 cm is 20% of total, it is possible to estimate a diagnosis of 580 NF-PanNEN ≤2 cm per year only in Europe. Worldwide the estimation of new NF-PanNEN ≤2 cm is around 29,840 cases in 5 years. The number of expected patients to be enrolled in the ASPEN study is at least 1,000 during the study period.

### Inclusion Criteria

Inclusion criteria include:

- Age > 18 years
- Individuals with asymptomatic sporadic NF-PanNEN ≤2 cm
- Diagnosis has to be proven by a positive fine-needle aspiration (FNA) or by the presence of a measurable nodule on high-quality imaging techniques that is positive at ^68^Gallium DOTATOC-PET
- Patients who undergo surgery for NF-PanNEN ≤2 cm within 12 months. In these cases, diagnosis has to be proven by histological confirmation of NF-PanNEN
- Informed consent.

### Exclusion Criteria

Exclusion Criteria include:

- NF-PanNEN > 2 cm of diameter
- Presence of genetic syndrome (Multiple Endocrine Neoplasia [MEN] type 1 syndrome, Von Hippel–Lindau [VHL] disease, Neurofibromatosis)
- Specific symptoms suspicious of a clinical syndrome related to hypersecretion of bioactive compounds or unspecific symptoms (functioning PanNEN).

### Diagnostic Work-Up

**Figure 1:**
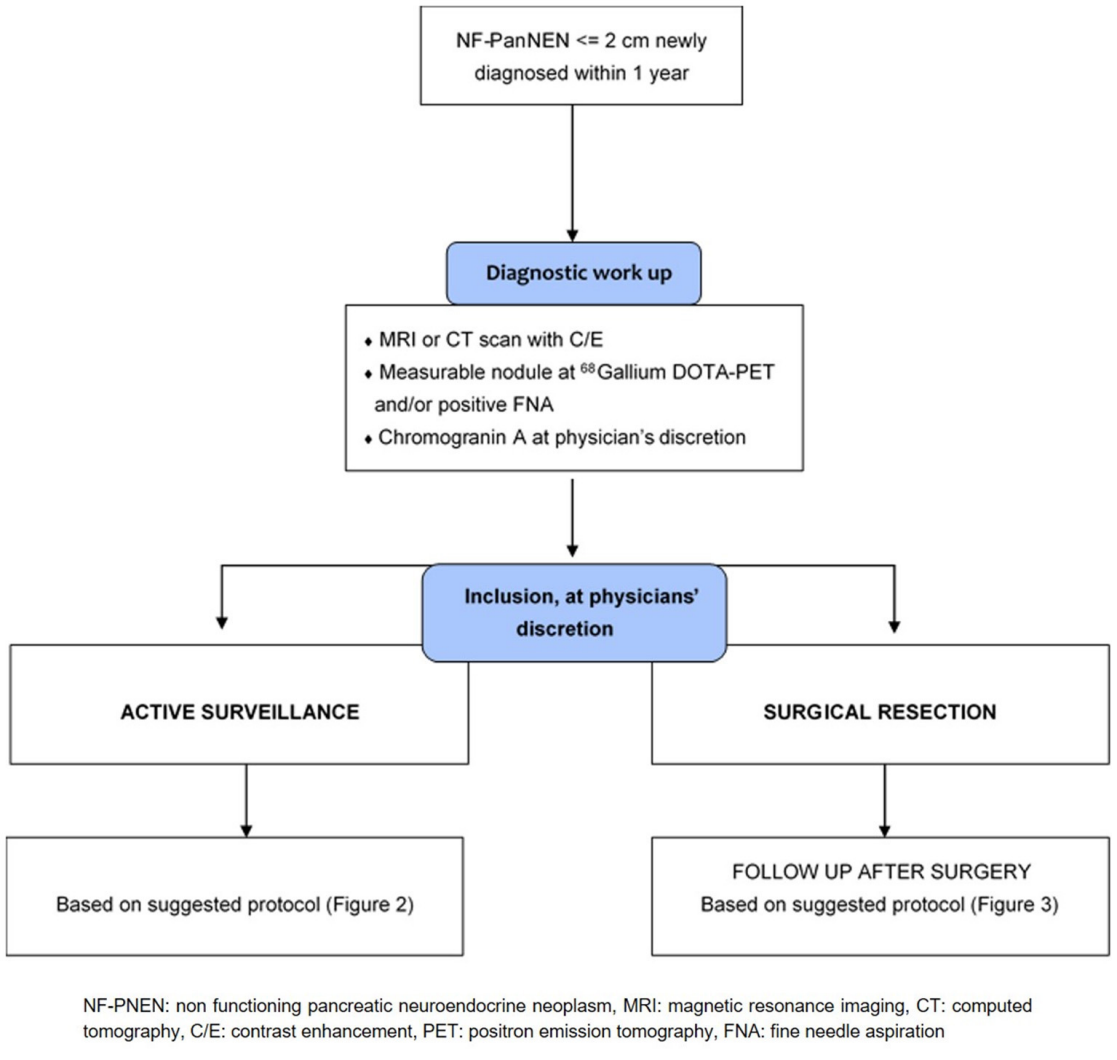
Diagnostic work up at inclusion.

Diagnostic work-up chart is provided in Figure 1. Every patients should be submitted before inclusion to diagnostic workup to characterize the neoplasm and to rule out the presence of other lesions (i.e., ductal adenocarcinoma, accessory spleen, solid serous cystadenoma). This work-up should have been performed no more than 12 months prior to inclusion. A high quality cross-sectional imaging study, either Computed Tomography (CT) or Magnetic Resonance Imaging (MRI) is mandatory. Diagnosis has to be proven by a positive fine-needle aspiration (FNA) or by the presence of a measurable nodule on high-quality imaging technique (CT or MR) that is positive at ^68^Gallium DOTATOC-PET scan. Patients who undergo surgery for NF-PanNEN ≤2 cm within 12 months can also be enrolled, in these cases, diagnosis has to be proven by histological confirmation of NF-PanNEN.

### Treatment Allocation

**Figure 2:**
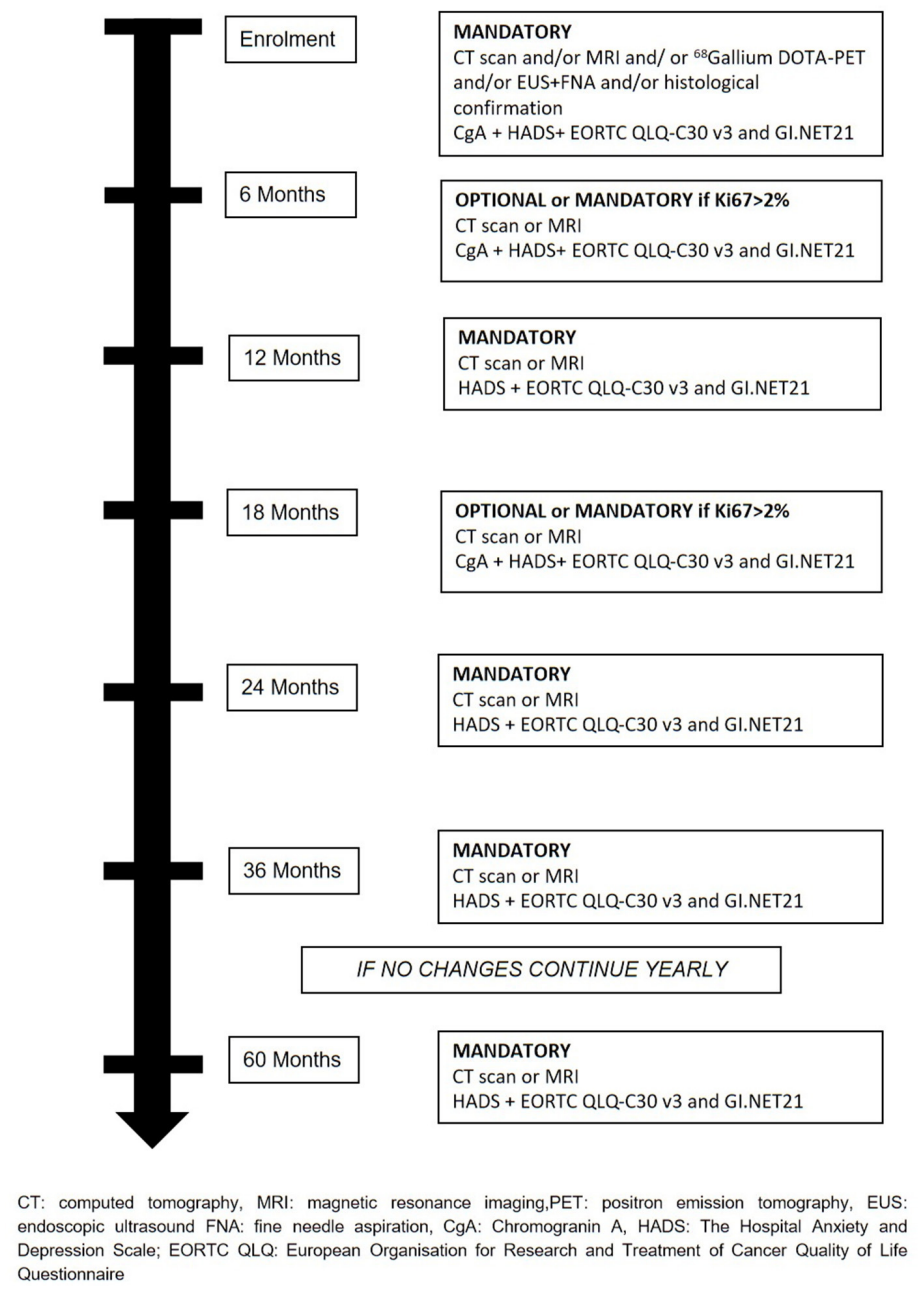
Suggested scheme of active surveillance for sporadic asymptomatic NF-PanNEN ≤2 cm.

**Figure 3:**
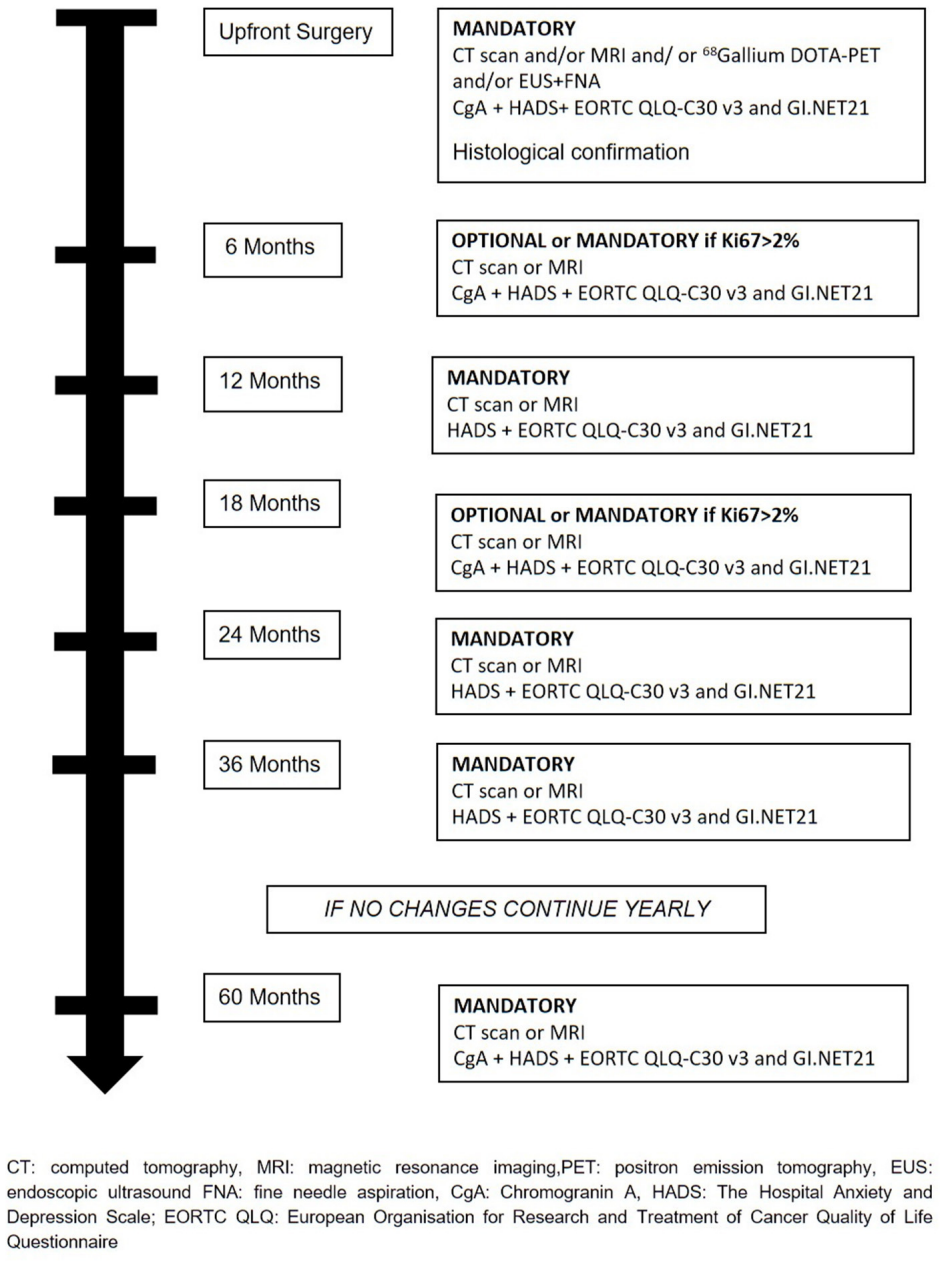
Suggested scheme after surgical resection for sporadic asymptomatic NF-PanNEN ≤2 cm.

The treatment will be decided at the hospital where patients are enrolled and all therapeutics decision will be decided and coordinated by the treating physicians. Recommended surveillance strategy consists of imaging studies (CT or MR), every 6 months for the first 2 years and yearly thereafter for 2 years in the absence of significant changes on imaging or symptoms appearance. During surveillance, a high-quality imaging technique (CT or MRI) is mandatory at least every 12 months or every 6 months if Ki67 is > 2%. Determination of Chromogranin A (CgA) during follow-up is at physician's discretion. During active surveillance, the treating physicians are responsible for patient management and decision-making. If follow-up parameters change during observation, the decision for further investigations, surgery, or an intensified follow-up schedule is at the discretion of the treating physicians (Figure 2). If surgical resection is warranted, timing and type of resection is established by treating physicians. Suggested scheme of follow up after surgery is depicted in Figure 3. If during surveillance NF-PanNEN size increases >2 cm and surgery is not performed, the reason should be stated. In this case, patient is not excluded and follow-up will continue regularly. Patients are asked to fill a questionnaire regarding the burden of NF-PanNEN (Hospital Anxiety and Depression Scale—HADS) and two questionnaires regarding quality of life of patients with NF-PanNEN (EORTC QLQ-C30—version 3.0 and EORTC QLQ-GI.NET 21). All three modules are administered at initial diagnosis, during surveillance and during follow-up after surgery at each visit. All data are recorded by treating physician on a specific web-based site.

### Statistical Analysis

Depending on distributional properties of the observed variable, percentages, means ± standard deviation (SD), or medians with interquartile ranges (IQR) will be reported. Statistical significance will be assessed with use of the Student's *t*-test for normally distributed continuous data; either the chi-square test for categorical data (with Yates' correction when appropriate) or Fisher exact test for categorical data; and the median test for non-normally distributed continuous data. All reported *p*-values will be two-sided and a value < 0.05 will be considered significant. For the primary endpoints, univariate comparisons will be conducted, to identify individual patient and NF-PanNEN risk factors for progression/recurrence. Outcomes will be evaluated in the intention-to-treat population based on treating physician-assessed tumor progression/recurrence. Survival analysis techniques and Cox regression with time-dependent recurrent covariates measures will be applied. Progression/recurrence is defined according to Response Evaluation Criteria in Solid Tumors (RECIST) version 1.0 criteria (24). In the surveillance group progression is defined as the appearance of distant metastases and/or local signs of invasiveness (i.e., vascular or nearby organs invasion). The mere tumor size increasing will be not considered a sign of progression unless it reaches >2 cm of maximum diameter. Rate of expect events is 0–10% for the two groups. Multivariate survival analysis will only be performed if the number of events will be >30.

## Discussion

From 2008 to 2012, the incidence of PanNEN raised from 0.4/100,000 to 0.8/100,000 inhabitants (7). This substantial increased is partially explained by the high number of diagnoses of small incidentally discovered NF-PanNEN that have become increasingly recognized entities in the last decades. Despite these figures show that small NF-PanNEN is still a relatively uncommon entity, several evidence support the hypothesis that their real occurrence is much higher. This was demonstrated by Canto et al. (26) who reported an incidental detection of a small NF-PanNEN in the 1% of asymptomatic patients who were enrolled in a screening program since their high-risk of developing pancreatic cancer. In another study (27) it was also found a prevalence of 4% of small NF-PanNEN that were incidentally detected by the pathologist in surgical specimen after pancreatic resection performed for a diagnosis other than neuroendocrine disease. As far as the diagnosis of these small nodules become even more frequent, it is of paramount importance to understand which should be their best management. This depends essentially by an adequate weighting of risks of over- and under-treatment since the natural evolution of these small lesions is largely unknown. Localized NF-PanNEN has been traditionally treated with radical surgical resection regardless their size. Recently, a conservative management with imaging-based follow-up has been emerging as a good alternative at least for selected patients (15–20). Two systematic reviews (20, 21) have evaluated the literature comparing surveillance and surgery in the management of asymptomatic, sporadic, small NF-PanNENs. Active surveillance seems to be safe at least in a mid-term follow-up. According to current evidence-based international guidelines draft by the ENETS society (2), a “*wait and see*” approach can be considered for asymptomatic PanNEN with a diameter of 2 cm or smaller. Similarly, recent recommendations by the North America Neuroendocrine Tumor Society (NANETS) support initial observation for asymptomatic NF-PanNEN smaller than 1 cm (28). Others have questioned the safety of a watchful strategy showing that the overall survival is significantly higher in patients who underwent surgery compared to those who are observed (22) and the guidelines for management of small NF-PanNENs are not yet well accepted since the rate of formal resections is high (29, 30). This skepticism is probably due to the lack of prospective studies and robust data on long-term follow-up. The ASPEN study is the first prospective multicentric study investigating the best management for small asymptomatic NF-PanNEN ≤2 cm. In this study, the natural history of small NF-PanNEN is prospectively evaluated in a multicentric setting, allowing the treating physicians to choose the best therapeutic option for each single patient. The option of designing a randomized clinical trial has been carefully evaluated before planning the study. Nevertheless, this possibility has been ruled out since the important differences in terms of possible side effects between the two types of treatment. On the other hand, the presence of strict inclusion and exclusion criteria as well as the absence of well-known characteristics of aggressiveness other than tumor size, may reduce the bias related to physicians' choice of patients' management. It has been reported that the most important factor leading to a surgical intervention of small NF-PanNEN is patients' preference (20, 30), although the real impact of follow-up on patients' anxiety and quality of life is unknown. One possible limitation of the current protocol is the relatively short period of follow-up given the possible slow evolution of these lesions. Nevertheless, the authors' aim is to continue the follow-up of these patients also after the end of the study providing a specific amendment of the protocol.

This prospective study aims also to clarify this important issue by constantly evaluating the psychological and physical burden on patients of the two different types of approaches. The most appropriate timing of observation is another matter of debate. In the current protocol, a high-quality imaging evaluation by either CT scan or MR on a yearly-basis is mandatory, whereas, a stricter observation schedule is at physicians' discretion. The primary endpoint is to evaluate any difference in terms of progression free survival that is another important strength of this prospective study. Previous retrospective studies based on large series, failed to address this important issue limiting the analysis on the overall survival (20, 22). In the ASPEN study, in order to improve study quality as much as possible, a large group of different institutions from more than 16 countries has been involved. This offers the opportunity not only to include a large number of patients but also to have a wider heterogeneity of management.

In conclusion, the ASPEN study is a multicenter prospective observational study investigating different management (active surveillance vs. surgery) of asymptomatic NF-PanNEN ≤2 cm. This study aims to provide evidence on the safety of an observational management of these tumors evaluating also the impact on patients' anxiety and quality of life. If this hypothesis is confirmed, a watchful attitude toward these small lesions will be more accepted worldwide reducing the surgery-related risks and improving patients' outcomes.

## Study Status

The first patient was enrolled on 31th August 2017. At the time of protocol submission (August 2019), 41 centers were actively recruiting patients for the study and 480 out of 1,000 patients (48%) had been enrolled. Inclusion is according to schedule.

## Ethics Statement

The studies involving human participants were reviewed and approved by IRRCS San Raffale Scientific Institute Ethics Committee. The patients/participants provided their written informed consent to participate in this study.

## Author Contributions

All the authors contributed to the conception and design of the study. Analysis of the literature and drafting of the manuscript was performed by SP, FM, and MF.

## Conflict of Interest

The authors declare that the research was conducted in the absence of any commercial or financial relationships that could be construed as a potential conflict of interest. The reviewer GL declared a shared affiliation, though no other collaboration, with one of the authors MC to the handling Editor.

## References

[1] Klimstra DS Kloppell G La Rosa SRG editor. The 2019 WHO classification of tumors of the digestive system. In: 5th International Agency for Research on Cancer. Lyon (2019) p. 16.

[2] Falconi M Eriksson B Kaltsas G Bartsch DK Capdevila J Caplin M ENETS consensus guidelines update for the management of patients with functional pancreatic neuroendocrine tumors and non-functional pancreatic neuroendocrine tumors. Neuroendocrinology. (2016) 103:153–71. 10.1159/000443171 26742109 PMC4849884

[3] Jilesen APJ Van Eijck CHJ Busch ORC Van Gulik TM Gouma DJ Van Dijkum EJMN. Postoperative outcomes of enucleation and standard resections in patients with a pancreatic neuroendocrine tumor. World J Surg. (2016) 40:715–28. 10.1016/j.hpb.2016.01.283 26608956 PMC4746212

[4] Hashim YM Trinkaus KM Linehan DC Strasberg SS Fields RC Cao D. Regional lymphadenectomy is indicated in the surgical treatment of pancreatic neuroendocrine tumors (PNETs). Ann Surg. (2014) 259:197–203. 10.1097/SLA.0000000000000348 24253141 PMC4164305

[5] Kuo EJ Salem RR Population-level analysis of pancreatic neuroendocrine tumors 2 cm or less in size. Ann Surg Oncol. (2013) 20:2815–21. 10.1245/s10434-013-3005-7 23771245

[6] Vagefi PA Razo O Deshpande V McGrath DJ Lauwers GY Thayer SP. Evolving patterns in the detection and outcomes of pancreatic neuroendocrine neoplasms: The Massachusetts General Hospital experience from 1977 to 2005. Arch Surg. (2007) 142:347–54. 10.1001/archsurg.142.4.347 17438169 PMC3979851

[7] Dasari A Shen C Halperin D Zhao B Zhou S Xu Y. Trends in the incidence, prevalence, and survival outcomes in patients with neuroendocrine tumors in the United States. JAMA Oncol. (2017) 3:1335–42. 10.1001/jamaoncol.2017.0589 28448665 PMC5824320

[8] Crippa S Partelli S Zamboni G Scarpa A Tamburrino D Bassi C. Incidental diagnosis as prognostic factor in different tumor stages of nonfunctioning pancreatic endocrine tumors. Surgery. (2014) 155:145–53. 10.1016/j.surg.2013.08.002 24646958

[9] Birnbaum DJ Gaujoux S Cherif R Dokmak S Fuks D Couvelard A. Sporadic nonfunctioning pancreatic neuroendocrine tumors: prognostic significance of incidental diagnosis. Surgery. (2014) 155:13–21. 10.1016/j.surg.2013.08.007 24238123

[10] Bettini R Partelli S Boninsegna L Capelli P Crippa S Pederzoli P. Tumor size correlates with malignancy in nonfunctioning pancreatic endocrine tumor. Surgery. (2011) 150:75–82. 10.1016/j.surg.2011.02.022 21683859

[11] Haynes AB Deshpande V Ingkakul T Vagefi PA Szymonifka J Thayer SP. Implications of incidentally discovered, nonfunctioning pancreatic endocrine tumors: short-term and long-term patient outcomes. Arch Surg. (2011) 146:534–8. 10.1001/archsurg.2011.102 21576607 PMC3688044

[12] Cherenfant J Stocker SJ Gage MK Du H Thurow TA Odeleye M. Predicting aggressive behavior in nonfunctioning pancreatic neuroendocrine tumors. Surgery. (2013) 154:785–91. 10.1016/j.surg.2013.07.004 24074416

[13] Partelli S Gaujoux S Boninsegna L Cherif R Crippa S Couvelard A. Pattern and clinical predictors of lymph node involvement in nonfunctioning pancreatic neuroendocrine tumors (NF-PanNETs). JAMA Surg. (2013) 148:932–9. 10.1001/jamasurg.2013.3376 23986355

[14] Falconi M Bartsch DK Eriksson B Klöppel G Lopes JM O'Connor JM. ENETS consensus guidelines for the management of patients with digestive neuroendocrine neoplasms of the digestive system: well-differentiated pancreatic non-functioning tumors. Neuroendocrinology. (2012) 95:120–34. 10.1159/000335587 22261872

[15] Gaujoux S Partelli S Maire F D'Onofrio M Larroque B Tamburrino D Observational study of natural history of small sporadic nonfunctioning pancreatic neuroendocrine tumors. J Clin Endocrinol Metab. (2013) 98:4784–9. 10.1210/jc.2013-2604 24057286

[16] Lee LC Grant CS Salomao DR Fletcher JG Takahashi N Fidler JL Small, nonfunctioning, asymptomatic pancreatic neuroendocrine tumors (PNETs): role for nonoperative management. Surgery. (2012). 2012:965–74. 10.1016/j.surg.2012.08.038 23102679

[17] Jung JG Lee KT Woo YS Lee JK Lee KH Jang KT. Behavior of small, asymptomatic, nonfunctioning pancreatic neuroendocrine tumors (NF-PNETs). Medicine. (2015) 94:e983. 10.1097/MD.0000000000000983 26131843 PMC4504528

[18] Sadot E Reidy-Lagunes DL Tang LH Do RKG Gonen M D'Angelica MI. Observation versus resection for small asymptomatic pancreatic neuroendocrine tumors: a matched case-control study. Ann Surg Oncol. (2016) 23:1361–70. 10.1245/s10434-015-4986-1 26597365 PMC4798427

[19] Rosenberg AM Friedmann P Del Rivero J Libutti SK Laird AM. Resection versus expectant management of small incidentally discovered nonfunctional pancreatic neuroendocrine tumors. Surgery. (2016) 159:302–9. 10.1016/j.surg.2015.10.013 26547726

[20] Partelli S Cirocchi R Crippa S Cardinali L Fendrich V Bartsch DK. Systematic review of active surveillance versus surgical management of asymptomatic small non-functioning pancreatic neuroendocrine neoplasms. Br J Surg. (2017) 104:34–41. 10.1002/bjs.10312 27706803

[21] Sallinen V Le Large TYS Galeev S Kovalenko Z Tieftrunk E Araujo R. Surveillance strategy for small asymptomatic non-functional pancreatic neuroendocrine tumors - a systematic review and meta-analysis. HPB. (2017) 19:310–20. 10.1016/j.hpb.2016.12.010 28254159

[22] Chivukula SV Tierney JF Hertl M Poirier J Keutgen XM Operative resection in early stage pancreatic neuroendocrine tumors in the United States: are we over- or undertreating patients? Surgery. (2020) 167:180–86. 10.1016/j.surg.2019.04.061 31537303

[23] Zigmond AS Snaith RP. The hospital anxiety and depression scale. Acta Psychiatr Scand. (1983) 67:361–70. 10.1037/t03589-000 6880820

[24] Fayers PM Aaronson NK Bjordal K on behalf of the EQ of LG The EORTC QLQ-C30 scoring manual. Third Edition. Brussels: European Organisation for Research Cancer.

[25] Yadegarfar G Friend L Jones L Plum LM Ardill J Taal B. Validation of the EORTC QLQ-GINET21 questionnaire for assessing quality of life of patients with gastrointestinal neuroendocrine tumors. Br J Cancer. (2013) 108:301–10. 10.1038/bjc.2012.560 23322194 PMC3566824

[26] Canto MI Hruban RH Fishman EK Kamel IR Schulick R Zhang Z. Frequent detection of pancreatic lesions in asymptomatic high-risk individuals. Gastroenterology. (2012) 142:796–804. 10.1053/j.gastro.2012.01.005 22245846 PMC3321068

[27] Partelli S Giannone F Schiavo Lena M Muffatti F Andreasi V Crippa S. Is the real prevalence of pancreatic neuroendocrine tumors underestimated? A retrospective study on a large series of pancreatic specimens. Neuroendocrinology. (2019) 109:165–70. 10.1159/000499606 31117106

[28] Howe JR Merchant NB Conrad C Keutgen XM Hallet J Drebin JA. The North American neuroendocrine tumor society consensus paper on the surgical management of pancreatic neuroendocrine tumors. Pancreas. (2020) 49:1–33. 10.1097/MPA.0000000000001454 31856076 PMC7029300

[29] Mintziras I Keck T Werner J Fichtner-Feigl S Wittel U Senninger N. Implementation of current ENETS guidelines for surgery of small (≤2 cm) pancreatic neuroendocrine neoplasms in the german surgical community: an analysis of the prospective DGAV StuDoQ pancreas registry. World J Surg. (2019) 43:175–82. 10.1007/s00268-018-4751-2 30097704

[30] Partelli S Mazza M Andreasi V Muffatti F Crippa S Tamburrino D. Management of small asymptomatic nonfunctioning pancreatic neuroendocrine tumors: limitations to apply guidelines into real life. Surgery. (2019) 166:157–63. 10.1016/j.surg.2019.04.003 31109657

